# Bilateral Adrenalectomy in a Patient With Refractory Primary Aldosteronism Due to Adrenal Hyperplasia

**DOI:** 10.7759/cureus.24267

**Published:** 2022-04-19

**Authors:** Luis Marín-Martínez, Antonio J Ríos-Vergara, Georgios Kyriakos, Maria C Álvarez-Martín, Enrique Hernández-Alonso

**Affiliations:** 1 Endocrinology and Nutrition, Hospital General Universitario Santa Lucía, Cartagena, ESP

**Keywords:** hypokalemia, bilateral adrenalectomy, adrenal hyperplasia, primary aldosteronism, resistant hypertension

## Abstract

Primary aldosteronism (PA) is a frequent cause of secondary hypertension. The main cause of PA is bilateral adrenal hyperplasia, and treatment is usually medical with mineralocorticoid receptor antagonists (MRAs) such as spironolactone or eplerenone. In this paper, we present a rare clinical case of a middle-aged female with refractory arterial hypertension and hypokalemia that complementary medical tests confirmed PA due to bilateral hyperplasia, and despite a maximum dose of spironolactone and oral potassium supplements, there was no clinical response. Because of this, finally, the patient needed surgical treatment based on bilateral adrenalectomy, which was effective. This is unusual and poorly described in the medical literature.

## Introduction

Primary aldosteronism (PA) is characterized by autonomic aldosterone hypersecretion independent of stimuli for its regulation (renin-angiotensin-aldosterone system (RAAS), intravascular volume, or blood potassium concentration) [1,2]. More than 20% of cases with treatment-resistant hypertension are due to this clinical entity [3]. The principal cause of PA is bilateral adrenal hyperplasia (also called idiopathic adrenal hyperplasia (IAH)) (65%), followed by the aldosterone-producing adenoma (APA) (35%) [4]. A differential diagnosis should be made with other causes that can lead to hypertension and hypokalemia, the most frequent being the combination of essential hypertension with the use of diuretics that eliminate potassium, but there are more causes such as renal artery stenosis, Cushing’s syndrome, some forms of congenital adrenal hyperplasia (11-beta-hydroxylase or 17-alpha-hydroxylase deficiency), Liddle’s syndrome, and 11-beta-hydroxysteroid dehydrogenase type 2 deficiency [5]. We report an unusual clinical case of refractory PA to medical treatment that finally required bilateral adrenalectomy.

## Case presentation

A 58-year-old Caucasian woman with long-standing high blood pressure, heterozygous familial hypercholesterolemia, and type 2 diabetes who in the last three years had required several hospital admissions for metabolic alkalosis with hypokalemia attributed to abundant vomiting due to digestive pathology (in addition to the use of beta-agonists for intrinsic asthma) returned to the emergency department presenting dizziness, nausea, paresthesia, and chest discomfort secondary to a hypertensive crisis with a value of 230/110 mmHg on arrival. No pathological signs were found on physical examination. The main laboratory workup showed a pH of 7.48, bicarbonate of 33 mmol/L, and serum potassium level of 3 mEq/L. The EKG was normal.

She was admitted to the internal medicine department. A funduscopic examination was done that ruled out malignant hypertension. The laboratory tests were completed with autoimmunity, proteinogram, and complement studies without relevant findings except for elevated urine potassium levels (>20 mEq/L). A genetic study for Liddle’s syndrome was negative. Renovascular hypertension was ruled out with a Doppler ultrasound that showed normal renal parenchyma without dilatation of the collecting systems and a CT angiography of the renal arteries that excluded the presence of stenosis.

Subsequently, consultation was requested from our endocrinology service, and a complete hormonal study of secondary arterial hypertension revealed a normal thyroid profile, minimal urine elevation of norepinephrine with normal metanephrines, and normal basal cortisol. Further workup for hypokalemia showed that the plasma concentration of aldosterone was 15.9 ng/dL and that the plasma renin activity (PRA) was 0.4 ng/mL/hour, resulting in an aldosterone/renin ratio (ARR) of 39.75. In addition, chromogranin A (in the normal range) and enolase (in the upper limit) were determined (Table 1).

**Table 1:** Analytical parameters

Analytical parameters	Laboratory values	Laboratory reference intervals
pH	7.48	7.31-7.41
Bicarbonate	33 mmol/L	24-28 mmol/L
Serum potassium	3 mEq/L	3.5-5.5 mEq/L
Urinary potassium	>20 mEq/L	17-85 mEq/L
Aldosterone	15.9 ng/dL	0.7-15 ng/dL
Plasma renin activity (PRA)	0.4 ng/mL/hour	0.8-2.1 ng/mL/hour
Aldosterone/renin ratio (ARR)	39.75 ng/dL / ng/mL/hour	<30 ng/dL / ng/mL/hour

**Figure 1:**
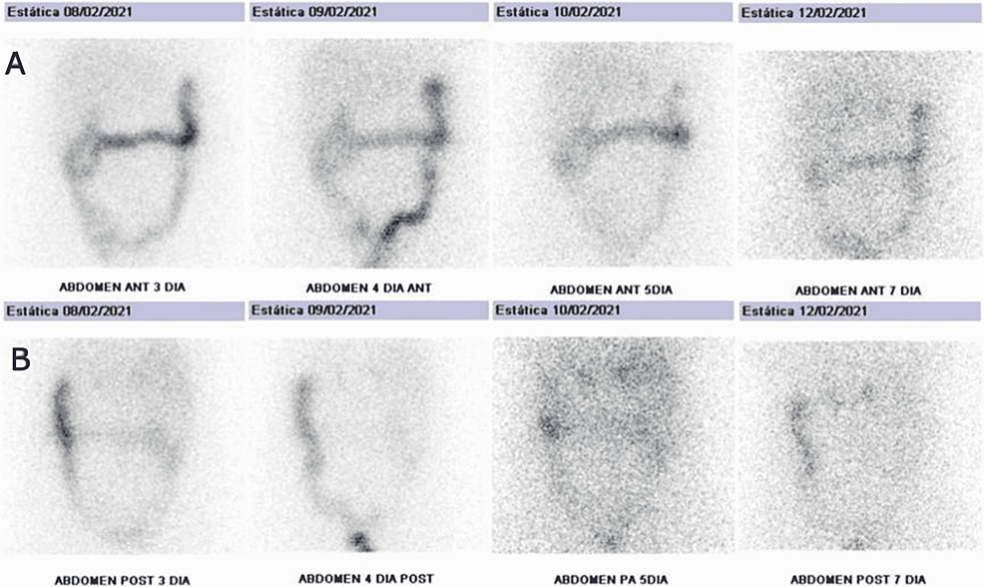
A: First phase of the scintigraphy with uptake predominantly in the colon. B: Second phase of the scintigraphy with uptake predominantly in the right adrenal gland.

**Figure 2:**
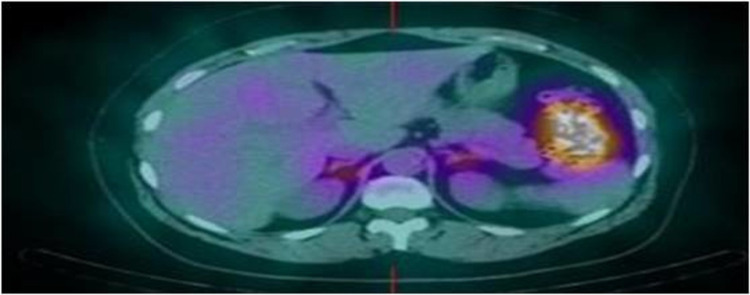
SPECT-CT: Bilateral metabolic activity increase in both adrenal glands.

The patient was discharged from the hospital after a symptomatic control with oral potassium supplements and spironolactone to finalize the study on an outpatient basis. In the ambulatory setting, norcholesterol scintigraphy and acquisition of SPECT-CT were performed. In the scintigraphy, after injecting a single dose of the tracer and taking dexamethasone simultaneously, the image corresponding to the fifth day showed increased uptake in two weak areas located anatomically in the adrenal glands, the right being somewhat better defined. The image obtained on the seventh day continued to show two foci, although with less activity and definition. These findings are compatible with idiopathic adrenal hyperplasia (IAH) (Figures 1, 2).

**Figure 3:**
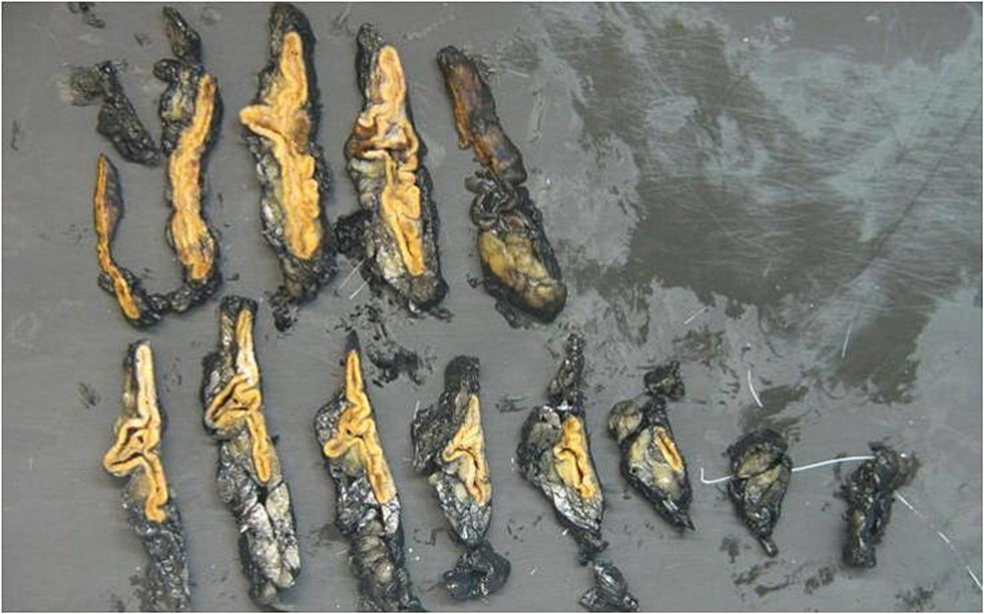
Right adrenal gland: surgical specimen weighing 15 g.

During her follow-up, despite sequentially increasing oral supplementation with potassium up to five tablets at each meal and spironolactone reaching a maximum dose (400 mg/day), hypokalemia and arterial hypertension persisted (she continued taking olmesartan 20 mg, doxazosin 8 mg, and amlodipine 10 mg), with progressive symptomatic worsening. So, the case was presented in a multidisciplinary clinical session, and after performing a regular CT scan that did not find any adenoma, right unilateral adrenalectomy was done once the patient accepted a surgical approach (Figure 3).

Regarding the histology of the adrenal cortex, it showed an increase in areas of small cells with a glomerular appearance and some micronodules of large cells with clear cytoplasm, sufficient findings to confirm the diagnosis of diffuse adrenal hyperplasia.

After the surgery, the patient had a partial improvement in blood pressure and serum potassium levels, but in subsequent medical checkups, she again required a higher intake of oral potassium and a further increase in the dose of spironolactone, for which it was necessary to complete the surgery doing left adrenalectomy in a second time. Six months after this second surgery, she is asymptomatic with normal blood pressure and serum potassium, without the need for continued medical treatment.

## Discussion

We present this clinical case to highlight the difficulty of diagnosing and treating a refractory PA due to bilateral adrenal hyperplasia that finally required bilateral adrenalectomy, mostly described in Cushing’s syndrome, but not in such cases [6,7].

We had some limitations to confirm the PA in our case. Although the ARR was highly suggestive, we were unable to perform a confirmation test. The refractory hypertension and hypokalemia of the patient made it impossible to suspend spironolactone and the other antihypertensive agents in order to obtain a reliable result.

Although the gold standard lateralization procedure remains bilateral adrenal vein sampling (AVS), scintigraphy with I131-norcholesterol is preferred as it is a less invasive test [1,8,9]. This technique allows the assessment of adrenal function and can be used to differentiate between APA and IAH (unilateral adrenal uptake most likely indicates APA, and bilateral uptake suggests IAH). The administration of dexamethasone, which suppresses adrenal cortical hormones, markedly improves the diagnostic accuracy of adrenal scintigraphy [10]. In addition, in our environment, scintigraphy can reduce waiting times compared to AVS.

In patients with primary aldosteronism due to bilateral adrenal disease, medical treatment with MRA is recommended as a first-line option. Spironolactone is used at the lowest effective dose, which usually is 100-200 mg/dL. In case of intolerance, eplerenone should be used [1,11].

If hypertension persists, it is necessary to add non-MRA antihypertensive medication (amiloride, thiazides, loop diuretics, calcium antagonists, angiotensin II receptor antagonists, and ACE inhibitors) [12]. Our patient always presented a poor control of arterial hypertension despite being treated with the maximum dose of spironolactone (400 mg/day), angiotensin II receptor antagonists, calcium antagonists, and beta-blockers. Furthermore, hypokalemia persisted despite supplements of potassium hydrogencarbonate.

In such cases, surgical treatment can be an option. It should be noted that there is no high-grade clinical trial evidence supporting lifelong MRA therapy in bilateral PA. Unilateral adrenalectomy, although does not cure patients with bilateral primary aldosteronism, can provide an improvement in blood pressure, the severity of hypokalemia, or the number of blood pressure medications needed [13,14]. However, this option did not improve primary aldosteronism in our patient, and we completed contralateral adrenalectomy.

Bilateral adrenalectomy for IAH is not recommended or supported by systematic evidence. However, there are anecdotal experiences described in selected cases. Although the procedure is curative for primary aldosteronism, in general, inducing iatrogenic primary adrenal insufficiency is not desirable. Thus, in situations where medical therapy for bilateral primary aldosteronism remains suboptimal and unilateral non-curative adrenalectomy is unable to improve the biochemical and clinical risk factors that suppose a high risk for cardiovascular event such a stroke, heart failure, and other complications, another adrenalectomy can be made [14].

## Conclusions

The diagnostic screening of primary aldosteronism consists of determining the ARR but requires careful operating conditions for its correct interpretation. If it is positive, a confirmation test should be performed, although on many occasions, as in our patient, it is not possible due to poor blood pressure control. When it comes to confirming lateralization, adrenal vein catheterization remains the gold standard, although it is an invasive test that is difficult to interpret. Instead, a norcholesterol scintigraphy may be performed. Bilateral hyperplasia is treated medically with mineralocorticoid receptor antagonists, and control is achieved in most cases with low-dose spironolactone (or eplerenone). The refractoriness to medical treatment of this patient is striking, even at the maximum dose that finally required bilateral adrenalectomy (practiced in Cushing’s syndrome but minimally referenced in the literature in primary aldosteronism due to bilateral adrenal hyperplasia). Likewise, the dissociation between the modest results of the complementary tests and the severity of the symptoms is paradoxical. Despite the above, the clinical evolution of the patient after the complete surgery was satisfactory, correcting blood pressure and potassium levels.
